# Role of Low Molecular Weight Heparin in the Management of Unexplained Recurrent Pregnancy Loss: A Review of Literature

**DOI:** 10.7759/cureus.10956

**Published:** 2020-10-15

**Authors:** Oluwatayo J Awolumate, Ayesha Kang, Rhutuja Khokale, Ivan Cancarevic

**Affiliations:** 1 Family Medicine, California Institute of Behavioral Neurosciences & Psychology, Fairfield, USA; 2 Internal Medicine, California Institute of Behavioral Neurosciences & Psychology, Fairfield, USA; 3 Neurology, California Institute of Behavioral Neurosciences & Psychology, Fairfield, USA

**Keywords:** unexplained recurrent pregnancy loss, low molecular weight heparin

## Abstract

Recurrent pregnancy loss remains a significant challenge in gynecological practice, accounting for about 2%-4% of pregnancies. In some patients, the etiology is unknown. Unexplained recurrent pregnancy loss (URPL) refers to the spontaneous loss of three or more consecutive pregnancies without an identifiable risk factor, accounting for about 40%-50% of pregnancy losses. The review aims to understand the role of low molecular weight heparin (LMWH) in the treatment of URPL. Articles for this review have been found in the PubMed database, and studies published more than ten years before the review excluded. The articles were reviewed to determine the effect of LMWH on live birth rates, reduced late pregnancy complications, and adverse drug reactions following its use. Many studies show improved live birth rates in women treated with LMWH compared to the control, while some studies show no improvement. There was no statistically significant difference in reducing late pregnancy complications, such as preeclampsia, intrauterine growth restriction, preterm labor, and low birth weight, in either study and control groups. Adverse drug reaction was rare among women treated with LMWH and, if present, was mild and self-limiting, thus making it a safe therapy. More studies, preferably large multicenter randomized controlled trials, need to be conducted on the use of LMWH to establish a consensus guideline on the treatment of URPL.

## Introduction and background

Recurrent pregnancy loss (RPL) remains a significant challenge in gynecological practice. It is not only emotionally devastating for expectant women but is also medically challenging. RPL is the spontaneous loss in the first trimester of three or more consecutive pregnancies with the same biological father [1,2]. It occurs in about 2%-4% of pregnancies [1,3]. Common causes include the uterine anatomic anomalies, endocrine/hormonal abnormalities, genetic/chromosomal abnormalities, and blood coagulation/platelet defects [1]. Many of these causes are treatable [1,3]. However, in about 40%-50% of pregnancy-loss cases, there is no identifiable cause [1,3]. As such, the term “unexplained recurrent pregnancy loss” (URPL) is the spontaneous loss of three or more consecutive pregnancies without an identifiable risk factor [2,3]. Studies revealed that placental thrombosis, vascular endothelial growth factor dysfunction, and maternal-fetal immunology play a role in women with URPL [3,4]. Proinflammatory changes such as increased Th1 to Th2 cytokine ratio and complement activation has been repeatedly demonstrated in these women [3,4,5]. There is also increasing knowledge of the association between methylenetetrahydrofolate reductase gene polymorphisms and URPL [5].

Developing guidelines for managing URPL is complicated, as the exact etiology is unknown [5,6]. Thus, empirical treatment with immunomodulators and drugs has been in practice with varying degrees of success [2,6]. These drugs include corticosteroids, human chorionic gonadotrophin, immunoglobulin, heparin, low-dose aspirin, paternal white cell immunization, progesterone, trophoblastic membrane infusion, tumor necrotic factor inhibitors, and vitamin supplementation [2,6].

Heparin was discovered in 1916, although the clinical use of its first therapeutic form, unfractionated heparin (UFH), started in the 1930s [7]. It is the most widely used clinical anticoagulant worldwide [7]. The various therapeutic forms of heparin-based drugs available in current clinical practice include UFH, low molecular weight heparin (LMWH), and synthetic heparins [7]. These heparin-based drugs are essential in treating thrombosis and embolisms and preventing thromboembolic phenomena [7]. Heparin works primarily by inhibiting thrombin (factor IIa) and factor Xa [7]. The ratios of anti-Xa activity to the anti-IIa activity of different heparins differ [7]. Heparin use is associated with the risk of bleeding, heparin-induced thrombocytopenia, and osteoporosis, requiring close monitoring [7]. The discovery of LMWH with a longer half-life, higher bioavailability, a more stable dose-response relationship, a better safety profile, and a reduced need for monitoring compared to UFH has made its use more attractive to patients and physicians [7,8]. LMWH has a shorter oligosaccharide/monosaccharide chain and higher anti-Xa/anti-IIa ratios than UFH [7]. However, it has the same efficacy in preventing and treating various coagulation disorders [4,7]. Because LMWH has both anticoagulative and anti-inflammatory effects, it is now widely used to treat recurrent pregnancy losses due to thrombophilia and nonthrombophilia, either alone or with other agents such as acetylsalicylic acid [4,5,8-12]. Outcomes such as live birth rate and the occurrence of pregnancy complications were some of the variables reported in some studies, with varying degrees of success [4,5,8-12].

Many observational studies and randomized control trials (RCTs) have been conducted concerning the use of LMWH alone or in combination with other agents in the treatment of URPL [1,2,5,6,9,12-14]. Some showed favorable outcomes in live births and reduced complications such as preeclampsia and preterm deliveries [10,11,15-18]. However, some were inconclusive [10,11,14]. This finding could be due to heterogeneity and limited sample sizes [10,11,15,16]. This article will explore whether the currently available literature supports recommending the use of LMWH for the treatment of URPL for improving pregnancy outcomes in terms of live birth rates, reduction in pregnancy complications, and side effects.

The articles used for this review were found in the PubMed database using the keywords “unexplained recurrent pregnancy loss” and “heparin.” Studies published more than ten years before this review were excluded, as well as studies where LMWH was used in combination with other drugs, were also excluded. The primary outcome reviewed was live birth rates while the reduction in late pregnancy complications and the presence of adverse drug reactions following the use of LMWH were the secondary outcomes. A full quality assessment of individual articles could not be performed due to limited access to some articles.

## Review

The efficacy of LMWH in the treatment of URPL has been studied widely. Most studies' outcome was live birth rates and reduced pregnancy complications such as preeclampsia, preterm delivery, and intrauterine growth restrictions. Participants were monitored for the risk of adverse drug reactions arising from the intervention. Table 1 shows the list of articles reviewed, and the basic characteristics of the population studied. All the participants are of the reproductive age group.

**Table 1:** The basic characteristics of the population in articles reviewed *Represent values not reported in the study. RCT, randomized control trial; RPL, recurrent pregnancy loss; BMI, body mass index in kg/m^2^

Author [Reference]	Year of Publication	Study Design	Mean Age (Years)	Mean Number of RPL	Mean BMI	Total Sample Size
Monien et al. [4]	2009	Prospective observational study	32.7 +/- 6.0	>2 =13 weeks	*	164
Yuksel et al. [17]	2014	Prospective Observational study	28 +/- 5	3	25.5 +/- 3	150
Cetin et al. [5]	2017	Retrospective study	28.40 +/- 5.20	3	*	121
Shaaban et al. [6]	2017	RCT	26.61 +/- 3.23	3	24.2 +/- 4.6	300
Xu et al. [12]	2018	Observation study	*	3	*	120
Pasquier et al. [10]	2015	Randomized (double-blind) Placebo control trial	32.7 +/- 5.2	3	23.9 +/- 4.4	258
De Jong et al. [9]	2014	RCT	*	2	*	1228
Han et al. [19]	2012	Retrospective study	*	3	*	72

Effect of LMWH on the live birth rate

Successful delivery of a live neonate is the goal of any pregnancy; thus, making live birth rate the primary indicator monitored in the articles reviewed. Monien et al. in a study of 164 women with early and late miscarriages having excluded thrombophilia, reported 83.8% of live births in women treated with LMWH [4]. Yuksel et al. in another prospective observational study of 150 women with a history of unexplained first-trimester pregnancy loss who received LMWH, reported a live birth of 85% in the LMWH group and 66% in the control group (p=0.007) [17]. Cetin et al. did a similar study on 120 women with unexplained recurrent miscarriages complicated with methylenetetrahydrofolate reductase gene polymorphism treated with LMWH and reported a live birth rate that was higher in patients with LMWH than in the control group (69.8% vs. 48.5% respectively, p: 0.015) [5]. Shaaban et al. in a randomized control trial of 300 participants with URPL, reported a significantly higher live birth rate of 65.7% vs. 36.2% in women treated with LMWH and folic acid group compared to the control group treated with folic acid as placebo (p = 0.001) [6]. Xu et al. in an hospital study of 120 URPL patients divided into two groups: control group (n = 60) and observation group (n = 60) reported an improvement in pregnancy success rate of patients in the observation group (90.00%) compared to control group (68.33%) (p < 0.05) [12].

Pasquier et al. in a multicenter randomized, double-blind placebo-controlled trial of 258 pregnant women with URPL having screened out thrombophilia cases reported no significant difference in the live birth rate of women who had LMWH compared to placebo 66.7% vs. 72.9%, respectively (p = 0.34) [10]. Schleussner et al. in a controlled, randomized multicenter trial of 449 women with URPL, also reported no statistically significant difference in the live-birth rates, 86.0% (185 of 215 women) vs. 86.7% (183 of 211 women) in the intervention and control groups, respectively (absolute difference, -0.7 percentage point [CI, -7.3 to 5.9 percentage points]) [13].

Overall, 1,562 patients participated in the seven studies. Patients in both groups were monitored until delivery or spontaneous termination of pregnancy. Five out of the seven articles reviewed for live birth rates, with 855 participants reported a significant improvement in the live birth rate of women who had LMWH compared to the control group [4-6,12-17]. In contrast, two of the articles with a total of 707 participants did not show any statistically significant difference in terms of live birth rate between the group who had LMWH and the control group [10,13].

**Figure 1:**
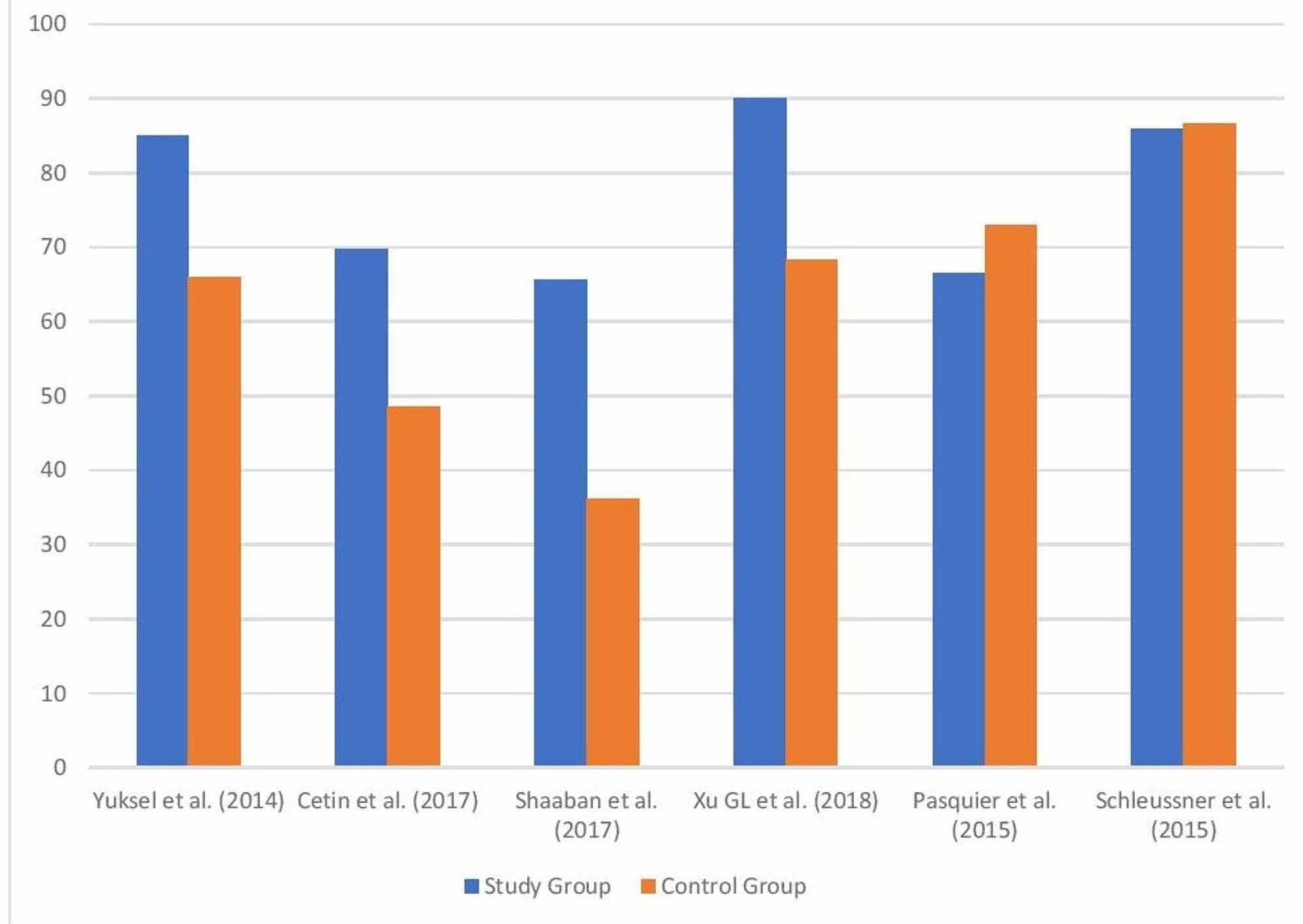
Bar chart comparing the live birth rates in the study group versus the control group

From the articles reviewed, shown in Figure 1, the use of LMWH conferred some improvement in the live birth rate of women with URPL. It should be used as soon as possible in the course of the pregnancy and continued until delivery. However, more studies still need to be done.

Effect of LMWH in reducing late pregnancy complications

There has been a high incidence of late pregnancy complications in women who had a history of recurrent pregnancy loss [18]. Some of the pregnancy complications associated with URPL include preterm contractions, preterm labor, preeclampsia, intrauterine growth restriction, low birth weight, and intrauterine fetal demise [18]. Studies have shown that the etiopathogenesis of the URPL could also be the reason for late pregnancy complications found in these women [18]. Thus, interventions effective in improving pregnancy outcomes in women with URPL will also invariably reduce the risk of late pregnancy complications. Late pregnancy complications as a secondary outcome were monitored in some articles reviewed.

Cetin et al. reported a significant reduction in the risk of congenital anomaly of 17.6% vs. 3.8% in the study and control groups [5]. However, there was no statistically significant difference in preeclampsia, preterm rate, and intrauterine growth restriction in the study group compared to the control group (p > 0.05) [5]. De Jong et al. also reported that obstetric complications such as preterm delivery, preeclampsia, intrauterine growth restriction, and congenital malformations were not significantly lower in the study and control groups [9]. Shaaban et al. reported a preeclampsia rate of 2.7% vs. 2.9% and fetal death rate of 13.7% vs. 27.5% in the study group vs. the control group, respectively, which were not statistically significant (p > 0.05) [6]. Schleusner et al. in another study on the use of LMWH for the treatment of URPL, reported some maternal and fetal complications following the use of LMWH [13]. These include three intrauterine fetal deaths, nine preeclampsia complicated by HELLP syndrome, and eleven intrauterine growth restriction due to placental insufficiency [13]. Li et al. observed no differences in birth weight or intrauterine growth restriction in both groups of pregnant women treated with LMWH for URPL compared to the control group [19]. Han et al. in the study conducted to determine the obstetric outcome of anti-inflammatory and anticoagulation therapy in women with recurrent pregnancy loss, reported a comparable incidence of preeclampsia 5.6% (n = 4), to that of the general population (2009 CDC data) (p > 0.05) [20]. There is also an increased incidence of HELLP syndrome (n=4) and preterm birth in the study group than the general population (21.7 versus 10.4%, p < 0.028) [20]. However, there was no statistically significant increase in early preterm birth (<34 weeks of gestation, 1.72%), small or large for gestation (10.6 and 4.3%, each), gestational diabetes mellitus (4.2%), and abruptio placentae (0 of 72) in the study group compared to general population data [20].

Overall, six studies monitored the occurrence or absence of late pregnancy complications in women who had LMWH for the treatment of URPL compared to those in the control group or the general populace. Five of these studies could not identify any increase or statistically significant changes in late pregnancy complications in the treatment group versus the control group or the general populace [5,6,9,13,19]. However, another study reported late pregnancy complications compared to the general populace [20]. LMWH has not increased late pregnancy complications in women with URPL and has not significantly reduced these complications.

Presence of adverse drug reaction following the use of LMWH

LMWH has an excellent safety profile as an anticoagulant compared to other heparin fractions [7]. However, patients on this drug should be monitored for possible complications such as bleeding, purpuric rash, thrombocytopenia, and osteoporosis [7,15]. Monitoring for adverse drug reaction is necessary as hematological changes in pregnancy could also increase adverse drug reactions using LMWH [21,22]. There could also be an increased risk of postpartum hemorrhage, a significant contributor to maternal morbidity and mortality worldwide in women using LMW heparin compared to the general population [21,22].

Monien et al. reported no severe side effects attributed to LMWH in women treated for URPL [4]. In another study, De Jong et al. reported that local skin reactions (pain, itching, swelling) to injection of LMWH were reported in almost 40% of patients treated who had received LMWH [9]. Yuskel et al. reported that maternal and neonatal side effects were not statistically significant among the study participants treated with LMWH than the control group [17]. Xu et al. in a study on the clinical efficacy of LMWH on unexplained recurrent pregnancy loss, reported an incidence of adverse drug reactions of 20.00% vs. 23.33% in women who had received LMWH compared to the control group (23.33%), which was not statistically significant with a p-value of > 0.05 [12]. Li et al. reported a sporadic occurrence of adverse effects in women who had LMWH for the treatment of URPL [19].

While most of the studies reported no adverse reactions attributed to LMWH in the treatment of URPL, a few studies reported some mild reactions [4,9,17,19]. Adverse reactions reported include local tissue reaction at the injection site, which in most cases were self-resolving and posed no significant risk [4,9,19]. Neither the dosage nor the route of administration of LMWH used in these studies was the same thus the need for a standardized dosage and route. It can be inferred from the articles reviewed for adverse drug reaction following the use of LMWH that its use is safe in the treatment of URPL, and its benefits far outweigh the possible risk of adverse drug reactions that could occur.

## Conclusions

Unexplained recurrent pregnancy loss, the spontaneous loss of three or more consecutive pregnancies without an identifiable risk factor, remains a significant challenge in gynecological practice. LMWH is now being used to treat URPL due to its anticoagulants and anti-inflammatory effect. Its use is associated with a substantial improvement in live birth rates in most studies reviewed with a few inconclusive. There was no significant improvement in late pregnancy complications in women treated with LMWH than the control group. Adverse drug reactions were rare among women treated with LMWH and, if present, it is usually mild and self-limiting, thus making it a safe therapy. More studies, preferably large multicenter randomized control trials, need to be done on the use of LMWH to establish a consensus guideline on the treatment of URPL.
